# Snakebite frequencies and envenomation case management in primary health centers of the Bobo-Dioulasso health district (Burkina Faso) from 2014 to 2018

**DOI:** 10.1093/trstmh/trab146

**Published:** 2021-09-28

**Authors:** Rabila Bamogo, Massamba Thiam, Achille Sindimbasba Nikièma, Fabrice Anyirekun Somé, Youssouph Mané, Simon Péguédwindé Sawadogo, Bazoumana Sow, Abdoulaye Diabaté, Youssouph Diatta, Roch Kounbobr Dabiré

**Affiliations:** Institut de Recherche en Sciences de la Santé (IRSS), Direction Régionale de l'Ouest, BP 545 Bobo Dioulasso 01, Burkina Faso; Université Cheikh Anta Diop, Ecole Doctorale en Sciences de la Vie, de la Santé et de l'Environnement, Faculté des Sciences et Techniques BP : 5005, Dakar, Sénégal; Université Cheikh Anta Diop, Institut Fondamental d'Afrique Noire (IFAN), Laboratoire de Zoologie et des Vertébrés terrestres de Dakar, BP 206, Dakar, Sénégal; Université Cheikh Anta Diop, Institut Fondamental d'Afrique Noire (IFAN), Laboratoire de Zoologie et des Vertébrés terrestres de Dakar, BP 206, Dakar, Sénégal; Institut de Recherche en Sciences de la Santé (IRSS), Direction Régionale de l'Ouest, BP 545 Bobo Dioulasso 01, Burkina Faso; Institut de Recherche en Sciences de la Santé (IRSS), Direction Régionale de l'Ouest, BP 545 Bobo Dioulasso 01, Burkina Faso; Institut de Recherche pour le Développement (IRD) de Dakar, BP 1386 CP 18524, Dakar, Sénégal; Institut de Recherche en Sciences de la Santé (IRSS), Direction Régionale de l'Ouest, BP 545 Bobo Dioulasso 01, Burkina Faso; Institut de Recherche en Sciences de la Santé (IRSS), Direction Régionale de l'Ouest, BP 545 Bobo Dioulasso 01, Burkina Faso; Institut de Recherche en Sciences de la Santé (IRSS), Direction Régionale de l'Ouest, BP 545 Bobo Dioulasso 01, Burkina Faso; Université Cheikh Anta Diop, Institut Fondamental d'Afrique Noire (IFAN), Laboratoire de Zoologie et des Vertébrés terrestres de Dakar, BP 206, Dakar, Sénégal; Institut de Recherche en Sciences de la Santé (IRSS), Direction Régionale de l'Ouest, BP 545 Bobo Dioulasso 01, Burkina Faso

**Keywords:** envenomation, epidemiology, frequencies, Hauts-Bassins, snakebites

## Abstract

**Background:**

Snakebite envenomation is a significant public health problem in Burkina Faso. Our study describes the epidemiological and therapeutic aspects of snakebite cases at primary health centers in Houet Province, which is located in the western area of Burkina Faso.

**Methods:**

We conducted a retrospective study of 664 snakebite cases occurring at 10 primary health centers in Houet Province from January 2014 to December 2018. Data were collected from the patient consultation recording database registry system.

**Results:**

Affected individuals had a male/female ratio of 1.31. The lowest annual incidences (0.02 [95% CI –0.01 to 0.05] and 0.24 [95% CI 0.05 to 0.43]) were observed in the urban primary health centers of Bolomakoté and Sarfalao, respectively. Rural primary health centers in Nasso in 2016 and in Soumousso in 2014 had the highest annual incidence (13.80 [95% CI 7.59 to 20.00] and 3.92 [95% CI 2.99 to 4.86], respectively). Of the 664 registered snakebite victims, none received antivenom immunotherapy treatment.

**Conclusion:**

Our study shows that snakebite envenomation incidents are common at the 10 primary health centers in Houet Province. Furthermore, despite the lack of antivenom and often inadequate treatment at these primary health centers, they remain the first point of care for snakebite victims.

## Introduction

Snakebite envenomation is a significant problem affecting all the continents of the world, in particular Africa, where it represents a major public health problem. Moreover, it is a problem often neglected by scientists, donors and international agencies as those affected are largely ‘people without a voice’. For example, in March 2017 at its 10th meeting, a subcommittee of the WHO Strategic and Technical Advisory Group on Neglected Tropical Diseases recommended that snakebite envenomation must be considered a neglected tropical disease.^1,2^ In West Africa, 16 low- and middle-income countries have 3500–5350 deaths from snakebite envenomation annually, or approximately 1.2 deaths per 100 000 individuals per year.^3^

In Burkina Faso, a landlocked country in West Africa, approximately 114 126 snakebites were reported during the 5 y from 2010 to 2014.^4^ This corresponds to >20 000 snakebite cases each year with a 3% case-fatality rate, as reported by the Ministry of Health. All sub-Saharan Africa countries are confronted with this problem, both in urban and rural areas.^5^ In Burkina Faso, snakebites are the fifth most common reason for admittance to Burkina Faso basic health facilities.^6^ Human-snake contact occurs mainly during field activities in high grass or during forest walks. However, human-snake contact can also occur around or in houses in regions with a high density of snakes, typically farming regions where grain attracts rodents, which, in turn, attract snakes.^7^ In Burkina Faso, snake species belong to six different families, of which *Elapidae* and *Viperidae* are the most venomous.^8^ Over the last 10 y, snakebite envenomation has gained more attention from health leaders and scientists, with decisions being made to improve snakebite envenomation management. Indeed, recent epidemiological studies have estimated snakebite incidence, and a system of data collection for tracking snakebite envenomation throughout the territory was established in 2010.^4,5,9^

In the health system of Burkina Faso, primary health center facilities are the first point of care for patients. It is at this primary level that patient care is first administered and decisions for potential referral to a higher level facility are made. Since 2015, the Ministry of Health has subsidized antivenom serums and made them available at public care centers for treating snakebite envenomation.^4^

The objective of the current study was to determine the frequency of snakebites in primary healthcare facilities and to identify the snake species responsible by conducting a prospective survey.

## Methods

**Figure 1.**
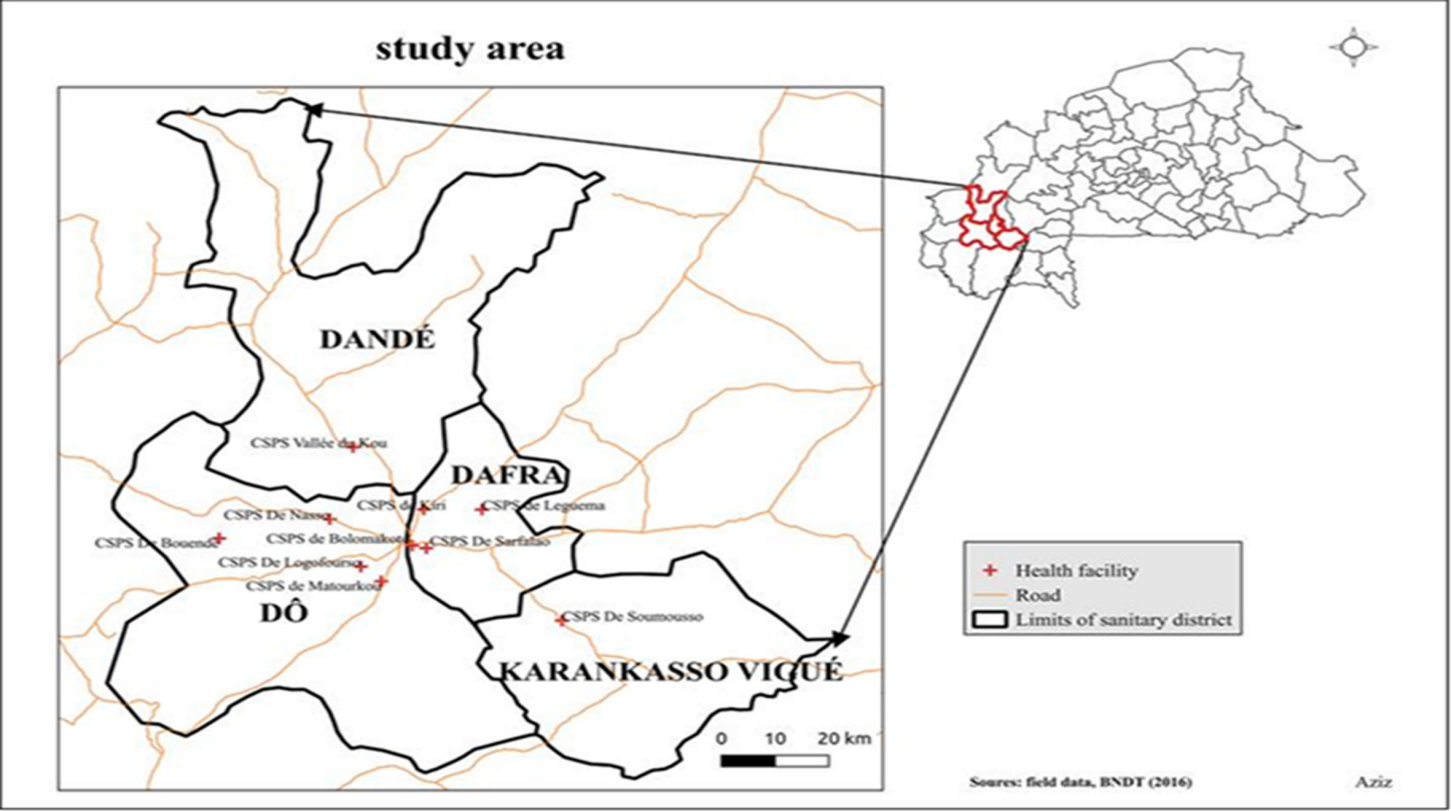
Survey site location in the health district of Bobo-Dioulasso (Hauts-Bassins). The red crosses represent the exact locations of each study site. The black bars represent the boundaries of each health district.

This study was conducted in Houet Province, located in the Hauts-Bassins administrative region of Bobo-Dioulasso (11°11′00″N; 4°17′00″W). This region is located in the western part of Burkina Faso, approximately 360 km from Ouagadougou, the capital city (Figure **1**). Houet Province covers a total area of 25.479 km^2^ (9.4% of the national territory). The population of Ouagadougou is approximately 1.7 million inhabitants. Climate is a Sudan climatic type and is characterized by a rainy season from May to October with relatively abundant rainfalls (the annual rainfall ranges from 1000 to 1200 mm) and a dry season from November to April. The annual average temperature is approximately 28ºC. Many individuals live in rural settings, where their access to healthcare depends on their proximity to the city Bobo-Dioulasso, where >80% of health services are located.

Ten primary health centers were selected for surveying: Bolmakoté, Bouende, Kiri, Leguema, Logofourousso, Matourkou, Nasso, Sarfalao, Soumousso and Valleé du Kou (Figure **1**). The main criteria justifying health center selection was a transect from the city to a rural setting that included a rice-growing area to a gallery forest. These criteria are representative of the ecological features of the region. The eco-climatic conditions of this region are suitable for snakes and human-snake contact seemed likely.

A retrospective survey was conducted, with data collected from the patient consultation recording database at primary health centers. These data included all patients referred for snakebite envenomation at the 10 primary health centers from January 2014 to December 2018. We used a survey card with a guide, a grid and a questionnaire. Annual incidence data were calculated using true population counts, with the total number of individuals in the village as the denominator, and are expressed with 95% CIs. Data were collected from patient health information and epidemiological surveillance centers in each district. Statistical analyses were performed using R 3.5. χ^2^ tests were used to compare differences between categories at a statistical significance of 5%.

A prospective survey of victims and their families was conducted from September 2018 to February 2019. The prospective survey aimed to better understand the circumstances of each snakebite and the fate of the patient bitten following care in a primary health center. Additionally, the survey also attempted to identify the snake responsible for the bite and to list the incriminated species in the list of snakebites occurring in the Hauts-Bassins region. For snake identification we relied on either the snake being physically brought to us, described by the victim or through species identification using photographs.^10^

A questionnaire was used to collect information about the nature and circumstances of each snakebite. This information is not typically collected by health centers. We also carried out focus group discussions to gain additional information from patients about their snakebites and the treatment they underwent.

## Results

### Population characteristics of those affected by snakebites

**Figure 2.**
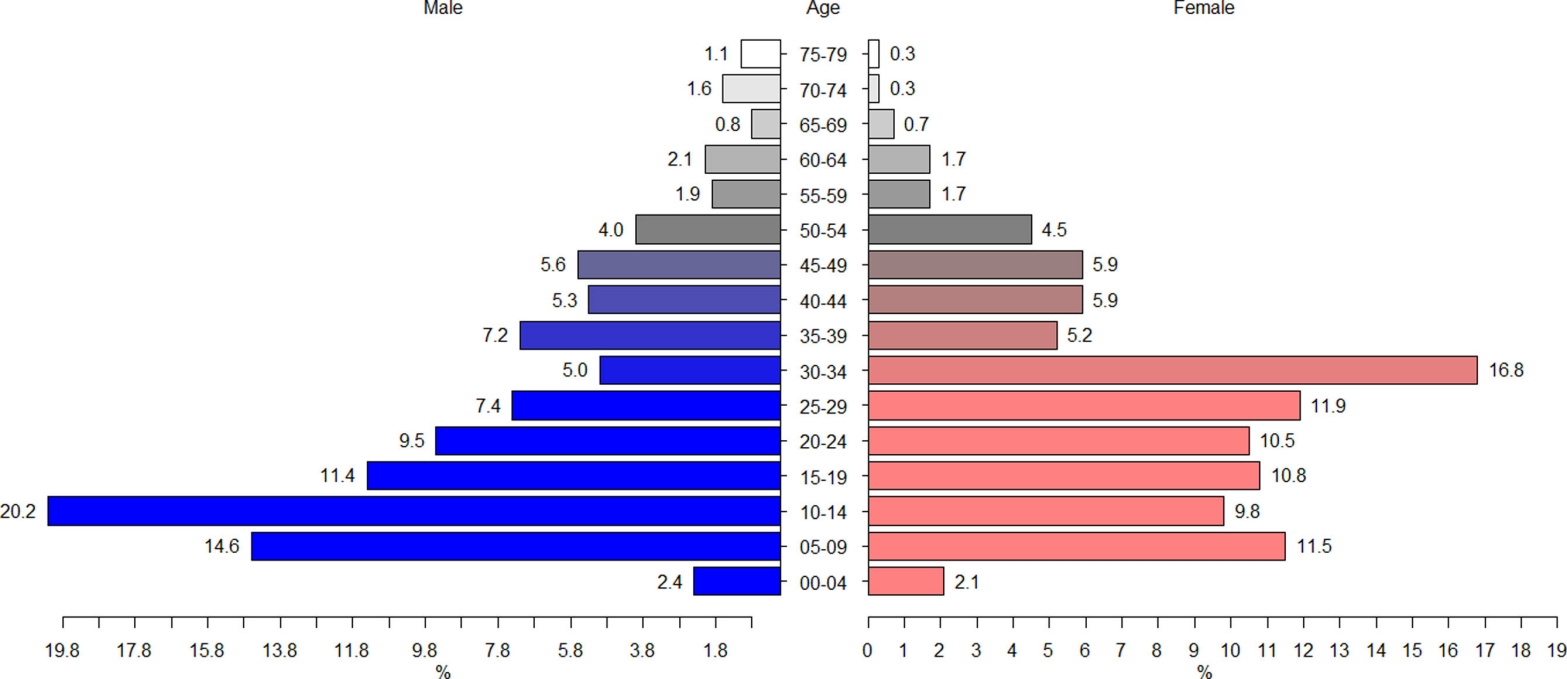
Pyramid of the ages of males and females of the population affected by snakebites. On the right is the frequency of snakebites for females by age group. On the left is the frequency of snakebites for males by age group.

A total of 664 snakebite cases were recorded from January 2014 to December 2018 in 10 primary health centers in the Hauts-Bassins region. Affected individuals had a male/female ratio of 1.31 (Figure **2**). Male patients aged 10–14 y were the most affected group, representing 20.2% of all male cases (Figure **2**). In females, those aged 30–34 y were the most affected group, representing 16.8% of all female cases.

### Incidence of snakebites in the Hauts-Bassins region during 2014–2018

The incidence of snakebites varied from 0.00 to 13.80 per 1000 individuals. The lowest annual incidences (0.02 [95% CI –0.01 to 0.05] and 0.24 [95% CI 0.05 to 0.43]) were observed in the urban primary health centers of Bolomakoté and Sarfalao, respectively (Table **1**). The rural primary health centers of Nasso in 2016 and Soumousso in 2014 had the highest annual incidence (3.92 [95% CI 2.99 to 4.86] and 13.80 [95% CI 7.59 to 20.00], respectively). Of the 664 surveyed individuals, 639 were from rural primary health centers. The mean bite rate (0.03) in urban primary health centers was significantly different to that in rural primary health centers (0.96; χ^2^=1135.5, df=1; p<0.0001). Over the entire 5-y period, peak snakebite incidence occurred during the rainy season. Rainy season incidents (448) occurred at approximately twice the rate of dry season incidents (216), a statistically significant difference (χ^2^=24.821, df=1; p>0.0001).

**Table 1.** Incidence of snakebites in the 10 primary health centers in the Hauts-Bassins region during 2014–2018

	Year									
Village	2014		2015		2016		2017		2018	
	Number of cases of snakebite Number of the population by year	Incidence rate per 1000 95% CI	Number of cases of snakebite Number of the population by year	Incidence rate per 1000 95% CI	Number of cases of snakebite Number of the population by year	Incidence rate per 1000 95% CI	Number of cases of snakebite Number of the population by year	Incidence rate per 1000 95% CI	Number of cases of Snakebite Number of the population by year	Incidence rate per 1000 95% CI
Bolomakoté	0	0	0	0	7	0.30	6	0.25	6	0.24
	22 025	[0.00–0.00]	22 752	[0.00–0.00]	23 504	[0.08–0.52]	24 258	[0.05–0.45]	25 036	[0.05–0.43]
Bouendé	1	0.24	8	1.88	4	0.92	9	2.00	12	2.50
	4096	[–0.23–0.72]	4251	[0.58–3.19]	4369	[0.02–1.81]	4511	[0.69–3.30]	4804	[1.08–3.91]
Kiri	5	0.75	4	0.58	15	2.11	3	0.41	10	1.32
	6649	[0.09–1.41]	6868	[0.01–1.15]	7093	[1.04–3.18]	7323	[–0.05–0.87]	7558	[0.50–2.14]
Leguema	12	1.57	0	0	3	0.37	11	1.31	4	0.46
	7653	[0.68–2.46]	7906	[0.00–0.00]	8164	[–0.05–0.78]	8428	[0.53–2.08]	8698	[0.01–0.91]
Logofourousso	0	0	0	0	7	1.35	12	2.25	5	0.91
	4852	[0.00–0.00]	5 013	[0.00–0.00]	5177	[0.35–2.35]	5344	[0.98–3.52]	5516	[0.11–1.70]
Matourkou	8	0.81	11	1.08	13	1.24	24	2.21	8	0.71
	9858	[0.25–1.37]	10 183	[0.44–1.72]	10 516	[0.56–1.91]	10 857	[1.33–3.09]	11 205	[0.22–1.21]
Nasso	9	0.70	30	2.25	19	13.80	33	2.32	15	1.02
	12 914	[0.24–1.15]	13 363	[1.44–3.05]	13 777	[7.59–20.00]	14 223	[1.53–3.11]	14 680	[0.50–1.54]
Sarfalao	0	0	0	0	1	0.02	3	0.04	2	0.03
	56 865	[0.00–0.00]	60 442	[0.00–0.00]	64 693	[–0.01–0.05]	66 789	[–0.01–0.10]	68 932	[–0.01–0.07]
Soumousso	68	3.92	60	3.38	36	1.96	22	1.16	28	1.43
	17 332	[2.99–4.86]	17 751	[2.52–4.24]	18 332	[1.32–2.61]	18 926	[0.68–1.65]	19 533	[0.90–1.96]
Vallé du Kou	26	1.05	26	1.02	27	1.03	23	0.85	28	1.00
	24 656	[0.65–1.46]	25 469	[0.63–1.41]	26 303	[0.64–1.41]	27 154	[0.50–1.19]	28 025	[0.63–1.37]
Overall incidence	129	0.77	139	0.80	132	0.73	146	0.78	118	0.61
	166 900	[0.64–0.91]	173 998	[0.67–0.93]	181 928	[0.61–0.87]	187 813	[0.65–0.90]	193 987	[0.50–0.72]

### Changes in annual incidence 

**Figure 3.**
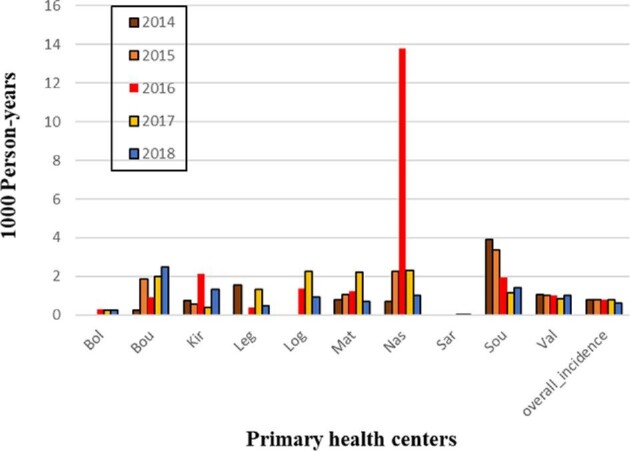
The evolution of the annual incidence by period and by primary health center. The x-axis represents the annual incidence by primary health center. The y-axis represents the change in incidence over time in years per 1000 persons. Bol, Bolomaké; Bou, Bouendé; Kir, Kiri; Leg, Leguema; Log, Logofourousso; Mat, Matourkou; Nas, Nasso; Sar, Sarfalao; Sou, Soumousso; Val, Vallée du Kou.

The primary health center in Nasso, a village of wooded savannah and dense forest, had an annual incidence of 13.80 per 1000 individuals in 2016. Similarly, the primary health center in Soumousso had an annual incidence of 1.96 per 1000 individuals in 2016. Interestingly, in 2018, both of these villages experienced significant decreases in incidence of 1.02 and 1.43, respectively (Figure **3**). The two urban primary health centers of Bobo-Dioulasso, Bolomakoté and Sarfalao, had very low annual snakebite incidence, ranging from 0 to 0.24 per 1000 individuals.

### Envenomation management 

**Figure 4.**
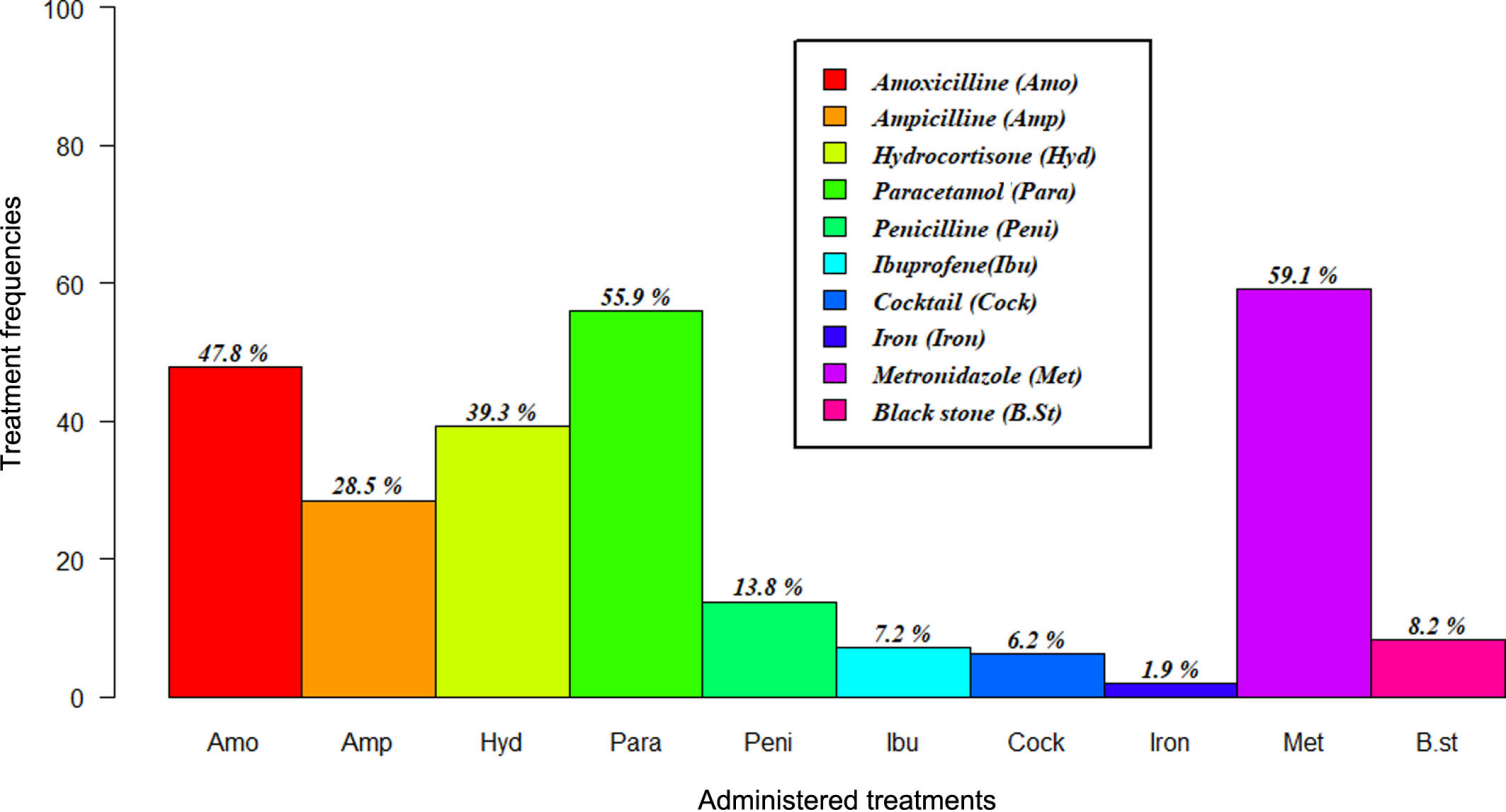
The proportions of drugs administered to patients bitten by snakes during their treatment.

Of the 664 snakebite victims, none received antivenom treatment in any of the 10 primary health centers surveyed from 2014 to 2018. Treatments were therefore symptomatic or prophylactic and consisted almost exclusively of metronidazole (59.1%) and paracetamol (55.9%). Amoxicillin was also administered in some cases (47.8%) (Figure **4**).

### Identification of snake species involved in biting incidents

**Figure 5.**
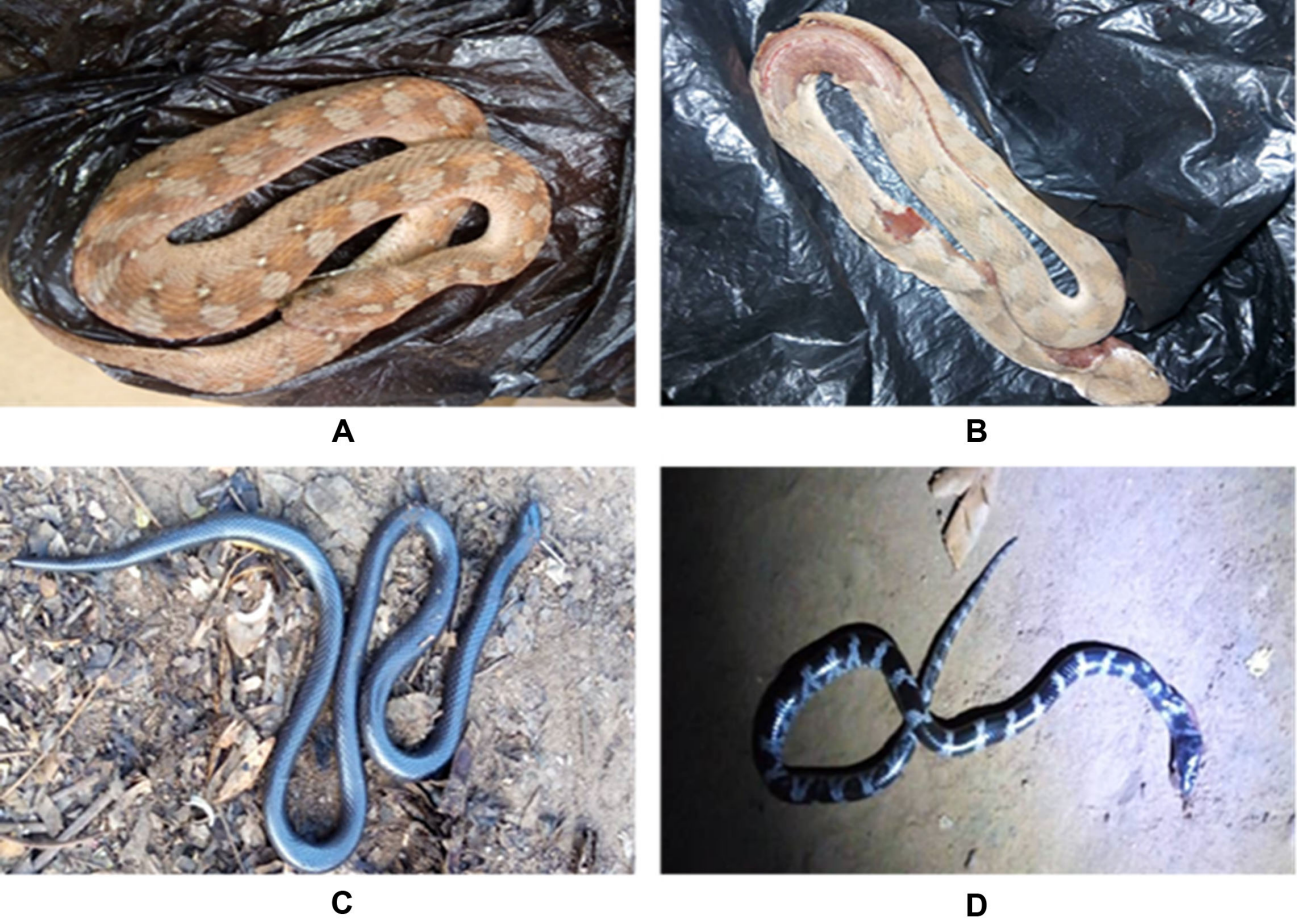
Distribution of the snake species involved in the biting cases recorded. (A) *Echis ocellatus* in the *Viperidae* family (village in Nasso); (B) *Echis ocellatus* in the *Viperidae* family (village in Matourkou); (C) *Atractaspis watsoni* in the *Lamprophiidae* family (village in Nasso); and (D) *Lycophidion semicinctum* in the *Colubridae* family (village in Nasso).

In our study, we received nine photographs and identified three large snake families (*Viperidae, Lamprophiidae* and *Colubridae*). From these three families we identified nine species: seven species of *Echis ocellatus* (*Viperidae*; Figure **5A,B**), one species of *Atractaspis watsoni* (*Lamprophiidae*; Figure **5C**) and one species of *Lycophidion semicinctum* (*Colubridae*; Figure **5D**).

## Discussion

### Characteristics of snakebite victims

The higher proportion of men involved in snakebite incidents may be explained by the larger number of men involved in agricultural and ranching activities that may expose them to more snakes. Similarly, women aged 30–34 y were the most affected age group of females (Figure **2**), possibly due to snake exposure during typical activities of shea nut picking, consumable shea caterpillar collection, cooking, wood cutting and farming activities. This may be especially true for housewives, who both cultivate and carry out housework. Boys aged 10–14 y were the most affected age group of males. This may be explained by the fact that these children still walk to school barefoot and often engage in risky behaviors such as reptile hunting, making them more vulnerable to snakebites. In addition, during the rainy season children typically work in the fields, helping their parents with cultivation or housing animals in barns. These activities may also increase their exposure to snakes.

Increased awareness about snakes as well as personal protective equipment, such as shoes, may help to reduce the frequency of snakebites in this age group. Rural areas and rice-growing zones are still conducive to snake proliferation and the risk of human-snake contact is much higher in these areas. Indeed, several studies have shown that rural populations are more vulnerable to snakebites than urban populations.^11–16^

These rural conditions may also explain the increased frequency of lower limb bites compared with upper limb bites. Regardless of the primary health center or village, 390 of the 556 individuals surveyed (70%) were bitten on their feet. Conversely, only 2% (11/556) and 0.17% (1/556) were bitten on the arm or forearm. This high proportion of leg bites may be due to a lack of agricultural personal protective equipment in low-income rural areas. Providing personal protective equipment to farmers and rural women could help to reduce the risk of snakebite exposure. We also encourage Burkina Faso and other countries to adopt agricultural policies that promote mechanization, as this may help to reduce manual farming and potential snakebite exposure.

The annual incidence of snakebites found in our study of the Hauts-Bassins region of Burkina Faso was higher in rural health centers than in urban health centers per 1000 individuals per year. This incidence in the current study is very low compared with that found in the same country by other authors in previous years, who reported an average of 100 snakebites per 100 000 individuals per year.^4^ We found that rural primary health centers typically observe at least one snakebite incident per 1000 individuals. A number of factors likely explains the differences between these two geographic health centers, as well as the overall low incidence in the urban primary health centers of Bolomakoté and Sarfalao. Poverty is one of the main risk factors for snakebites and the socioeconomic level in rural health center regions remains lower than the wealthier populations in urban cities.^17^ In addition, our case fatality rate (0.9%) was lower than those previously reported by Somé et al.^18^ and Gampini et al.^4^

We suspect destruction of natural snake habitats for housing or agriculture may explain the lower annual incidence observed from 2017 to 2018 in the villages of Nasso and Soumousso (Figure **3**), as these are semi-urban villages located approximately 10 to 25 km from the city of Bobo-Dioulasso. These practices have likely contributed to reducing accidental human-snake contact. Indeed, some ophidian species have ecological requirements that keep them away from ecosystems heavily modified by humans.^19^

### Envenom management in primary health centers

Antivenom immunotherapy is the only specific treatment for ophidian envenomation. Burkina Faso is the only sub-Saharan country where government subsidies allow for patients to receive a dose of antivenom (African Internal Standard Snake Antivenom Serum) at only 5% of the original price (<3US$).^4,20^ However, despite its low cost, antivenom is still not available in Burkina Faso health centers, with very few snakebite victims receiving antivenom immunotherapy. This is likely due to the continued use of traditional medicines and difficulties in distributing antivenom to rural health centers. This lack of antivenom immunotherapy in health centers, especially in outlying rural areas, likely increases the risk of death for snakebite victims. In our study we recorded 6 deaths from the 664 victims (0.9%). However, this may not be representative of the true case-fatality rate, as snakebite victims may have died before arriving at the treatment site. Of note among the six cases of death observed during our study, a young 21-y-old mother died following viper (*Echis ocellatus*) envenomization after being referred to a health center that had no antivenom available.

We recommend that the National Program for the Control of Neglected Tropical Diseases take the necessary steps to make antivenom immunotherapy available and accessible in Burkina Faso hospitals, as well as in primary health centers. We believe increased antivenom availability will significantly reduce case-fatality rates.

Symptomatic treatment often consists of analgesics, anti-inflammatory drugs and antibiotics. Chippaux demonstrated the importance of symptomatic treatments following snakebite envenomation.^21^ Patients experiencing fever, hypothermia or unexplained aggravation often receive a combination of penicillin G and an antibiotic (metronidazole or amoxicillin) administered in the face. These episodes are often related to nosocomial infections. Analgesics such as paracetamol are sometimes used to relieve pain. All treatments involved merely treating patient symptoms. Additionally, some patients (8.2%) benefit from black stone treatment; however, following a failure of this treatment, all patients end up going to the closest primary health center. The antibiotics used are either for prophylaxis or to treat secondary infections (Figure **4**). Other therapies such as coagulation factors, labile blood products and artificial respiration are sometimes used during symptomatic treatment; however, we did not observe any of these treatments in the current study. We did observe empirical treatments such as black stone treatment, which are of limited interest and likely add no clinical value, and may even cause harm. However, despite the controversial ineffectiveness of black stone treatment,^22^ it is still used in Burkina Faso communities for ophidian envenomation.

Our findings are consistent with the vicious cycle described by Chippaux in 2002.^23^ Specifically, the lack of a protocol for managing envenomation cases in combination with the lack of available antivenom, as well as ignorance of its use by health professionals, leads to a shortage of antivenom. We believe this vicious circle could be broken through financial support for antivenom distribution, particularly in rural areas, as well as the training of health workers on administering antivenom. These changes are undoubtedly a necessary condition for improving the snakebite management and reducing snakebite fatalities in Burkina Faso.

### Snake species identification

Few patients spontaneously bring the culprit snake to health centers, reducing the quality of patient care. Moreover, most snakebite victims are unable to provide any description of the culprit snake and relatively few patients are able to identify snakes by their local names. Based on local knowledge, many communities give the most venomous and most dangerous snakes local names such as ‘Fonfon-nii’ in the Dioula language. In our study we received nine photographs of culprit snakes (Figure **5A,B**). These photographs were taken by inexperienced personnel, justifying their poor quality. Although our snake picture sample size is insufficient, several authors have previously shown that vipers, notably *Echis ocellatus*, are a major contributor to snakebite envenomation in West Africa and particularly in Burkina Faso.^5,8,18,20,23,24^ We further encourage the National Program for the Control of Neglected Tropical Diseases to inform rural populations about the importance of bringing the killed culprit snake to the primary health center, as this will inform ophidian envenomation victim care.

### Study limitations

Our retrospective study has limitations consistent with that of other retrospective studies.^9^ Victim recall bias was a potential limitation, similar to all cross-sectional studies utilizing questionnaires of past events. Furthermore, nursing consultation records were not sufficiently detailed for understanding the victim's symptoms and the symptomatic treatment administered. In addition, most victims were unable to describe the snake that attacked them. Lastly, the reported data did not always contain relevant and useful information for implementing strategies to prevent or manage snakebites.

### Conclusion

Our study demonstrates that snakebite envenomation is relatively common in the 10 primary health centers of the Hauts-Bassins region and has a case fatality rate of 0.9% (6/664). Most snakebite cases were the result of a *Viperidae* family bite. Medical management of these snakebite victims was symptomatic and was dominated by metronidazole and paracetamol administration, which did constitute an adequate or recommended treatment regimen. Therefore, there is a critical need to develop and adopt snakebite management guidelines to sensitize vulnerable segments of the African population. Rural health centers would greatly benefit from making antivenom serum available to reduce the morbidity and mortality associated with snakebites. Furthermore, snakebite victims must be provided with immediate access to specific medical care, with a particular focus on antivenom therapy. Our current work enables future studies in other regions of Burkina Faso and Africa at large with similar ecological and environmental patterns. We hope these efforts will help improve snakebite epidemiological data and improve snake inhabitation maps, so as to reduce human-snake contact, accidental envenomation and death.

## Data Availability

The data is available in the link below: https://github.com/Rabila-Bamogo/Snake-data.git.

## Notes

**Authors’ contributions:** RKD and YD conceived the study; RKD, MT and RB designed the study protocol; RB, ASN, Fabrice Anyirekun Somé (FAS) and SPS carried out the investigations; RB and BS analyzed the data; RB and YM identified the species; RKD, MT and RB drafted the manuscript; RKD, AD, YM and YD critically revised the manuscript for intellectual content. All authors read and approved the final manuscript. RKD and RB are guarantors of the paper.
